# Incidence and Risk Factors Associated with Technique Failure in the First Year of Peritoneal Dialysis: A Single Center Retrospective Cohort Study in Southern China

**DOI:** 10.1186/s12882-022-02833-4

**Published:** 2022-06-11

**Authors:** Xiao Dong, Haishan Wu, Hongjian Ye, Chunyan Yi, Xiangwen Diao, Ruihua Liu, Haiping Mao, Fengxian Huang, Xueqing Yu, Xiao Yang

**Affiliations:** 1 Department of Nephrology, The First Affiliated Hospital, Sun Yat-Sen University, Guangzhou, China; 2 Key Laboratory of Nephrology, National Health Commission and Guangdong Province, Guangzhou, People’s Republic of China; 3 GuangdongProvincial People’s Hospital and Guangdong Academy of Medical Sciences, Guangzhou, China

**Keywords:** Technique failure, Peritoneal dialysis, Transferring to hemodialysis, Death

## Abstract

**Background:**

Technique failure is more likely to occur during the first 12 months after peritoneal dialysis (PD) initiation, which is a great challenge encountered in PD patients. The aim of this study was to investigate the incidence and risk factors associated with technique failure within the first year of PD patients in Southern China.

**Methods:**

Incident PD patients who were followed up for at least one year at The First Affiliated Hospital of Sun Yat-sen University from January 1, 2006 to December 31, 2015 were included. Technique failure was defined as transferring to hemodialysis (HD) for more than 30 days or death within the first year after start of PD. A competitive risk regression analysis was used to explore the incidence and risk factors of the technique failure.

**Results:**

Overall, 2,290 incident PD patients were included in this study, with a mean age of 48.2 ± 15.7 years, 40.9% female and 25.2% with diabetes. A total of 173 patients (7.5%) had technique failure during the first year of PD. Among them, the patient death account for 62.4% (*n* = 108) and transferring to HD account for 37.6% (*n* = 65). The main reasons for death were cardiovascular diseases (*n* = 32, 29.6%), infection (*n* = 15, 13.8%) and for conversion to HD were mechanical cause (*n* = 28, 43.1%), infection cause (*n* = 22, 33.8%). The risk factors for the technique failure included advanced age (HR 2.78, 95%CI 1.82–4.30), low body mass index (BMI < 18.5 kg/m^2^: HR 1.77, 95%CI 1.17–2.67), history of congestive heart failure (HR 2.81, 95%CI 1.58–4.98), or time on HD before PD ≤ 3 months (HR 1.49, 95%CI 1.05–2.10), peritonitis (HR 2.02, 95%CI 1.36–3.01);while higher serum albumin (HR 0.93, 95%CI 0.89–0.96) and using employee medical insurance to pay expenses (HR 0.47, 95%CI 0.32–0.69) were associated with reduced risk.

**Conclusions:**

Advanced age, poor nutritional status, history of HD or congestive heart failure, and peritonitis are related factors that increase the risk of technique failure in the first year of PD, while patients' type of medical insurance may also have an influence on early technique failure.

**Supplementary Information:**

The online version contains supplementary material available at 10.1186/s12882-022-02833-4.

## Introduction

The first year after the initiation of peritoneal dialysis (PD) is considered to be a high-risk period for PD failure. Studies have shown that about 40% of technique failures occurred in the first year of PD [1], which is a great challenge encountered in PD patients and a barrier to increased uptake of PD. The technique failure was defined as either transfering to hemodialysis (HD) for ≥ 30 days (death-censored) [2] or including death [3]. Due to the differences in the definition of technique failure, the technique failure rates during the first year of PD reported in different studies were varied from 12 to 26% [4]. The high technique failure rate in the early stage of PD directly offset the advantages of PD over HD in terms of economy and quality of life.

Australian and New Zealand Dialysis and Transplant Registry Centre (ANZDATA) showed that mechanical and other causes account for more cases during the first nine months of PD treatment [3]. A study on patients from the French Language Peritoneal Dialysis Registry (RDPLF) showed that patients treated by HD before PD and failed transplant patients had a higher risk of early PD failure when competing events were considered [5]. However, the key risk factors of technique failure within first year of PD treatment are still unclear. In the current study, we conducted a single center retrospective cohort study in Southern China to determine the incidence and risk factors associated with the technique failure within the first year of PD patients.

## Methods

### Design and population

This study was a single center retrospective cohort study. All adult patients (over 18 years old) who were catheterized and followed up for at least one year at The First Affiliated Hospital of Sun Yat-sen University from January 1, 2006 to December 31, 2015 were included.

### Study definitions

The technique failure was defined as transfer to HD for ≥ 30 days or death [3]. Peritonitis was diagnosed when at least 2 of the following were present: abdominal pain and/or cloudiness of dialysis effluent; white blood cell count in dialysis effluent > 100/μL, with > 50% polymorphonuclear leukocytes; and a positive culture from dialysis effluent [6]. Cardiovascular disease (CVD) was defined as a history of congestive heart failure, angina, myocardial infarction, coronary heart disease, cerebrovascular event, or peripheral vascular disease [7]. Cardiovascular (CV) death was defined as death due to congestive heart failure, acute myocardial infarction, cardiac arrhythmia, sudden cardiac arrest, cerebrovascular accident or peripheral vascular disease [8].

### Data collection

Prior to PD initiation, serum creatinine (Scr) was collected and baseline estimated glomerular filtration rate (eGFR) was calculated using the Chronic Kidney Disease Epidemiology Collaboration (CKD-EPI) creatinine equation [9]. Other baseline data during the first 1–3 months of PD initiation were also collected, including demographic data such as age, gender, primary cause of end stage renal disease (ESRD), presence of diabetes, history of CVD, education levels(higher education was defined as the completion of 12 years of compulsory education or above), medical insurance (the type of health insurance depends on the type of health insurance card that the patient pays for),complications, duration of RRT(renal replacement therapy), self-care ability (defined as patients can change the peritoneal dialysate on their own), occurrence of first peritonitis and body mass index (BMI). Biochemical data included levels of hemoglobin, serum albumin, creatinine, intact parathyroid hormone (i-PTH), albumin-corrected calcium, phosphorus, triglycerides, total cholesterol, high-density lipoprotein cholesterol (HDL-C) and low-density lipoprotein cholesterol (LDL-C).

### Clinical outcomes

The primary outcome was technique failure within the first year of PD initiation [2]. Secondary outcomes were cause-specific early technique failure which included transfering to HD and death.

### Statistical analysis

Categorical variables were expressed as frequency and proportion. Mean and standard deviation (SDS) were used to summarize normally distributed continuous variables. Median and interquartile range (IQR) were used to summarize continuous variables not following a normal distribution. χ^2^ test was used to compare the classified variables between groups, and Mann–Whitney test was used to test the skewed continuous variables. The risk factors of early technique failure were statistically analyzed by multivariate competitive risk regression model and renal transplantation was regarded as a competitive risk factor. The multivariate model included the related variables based on univariate analysis (*P* < 0.05). The interaction terms between covariables were tested in advance. Competitive risk regression analysis was also used to test secondary outcomes, and technique failures caused by death and transfering to HD were analyzed separately as target results. In these analyses, competing risks were transplant and the cause of other technique failures (death, transferring to HD). All covariates in the final model for the primary outcome were included in models for the secondary outcomes. In addition, we also generated a cumulative correlation function curve for each specific cause of technique failure (death, mechanical, infection, etc.) The data were analyzed by Stata/SE16.0, *P* < 0.05 was considered statistically significant.

## Results

### Study population and baseline characteristics

**Fig. 1:**
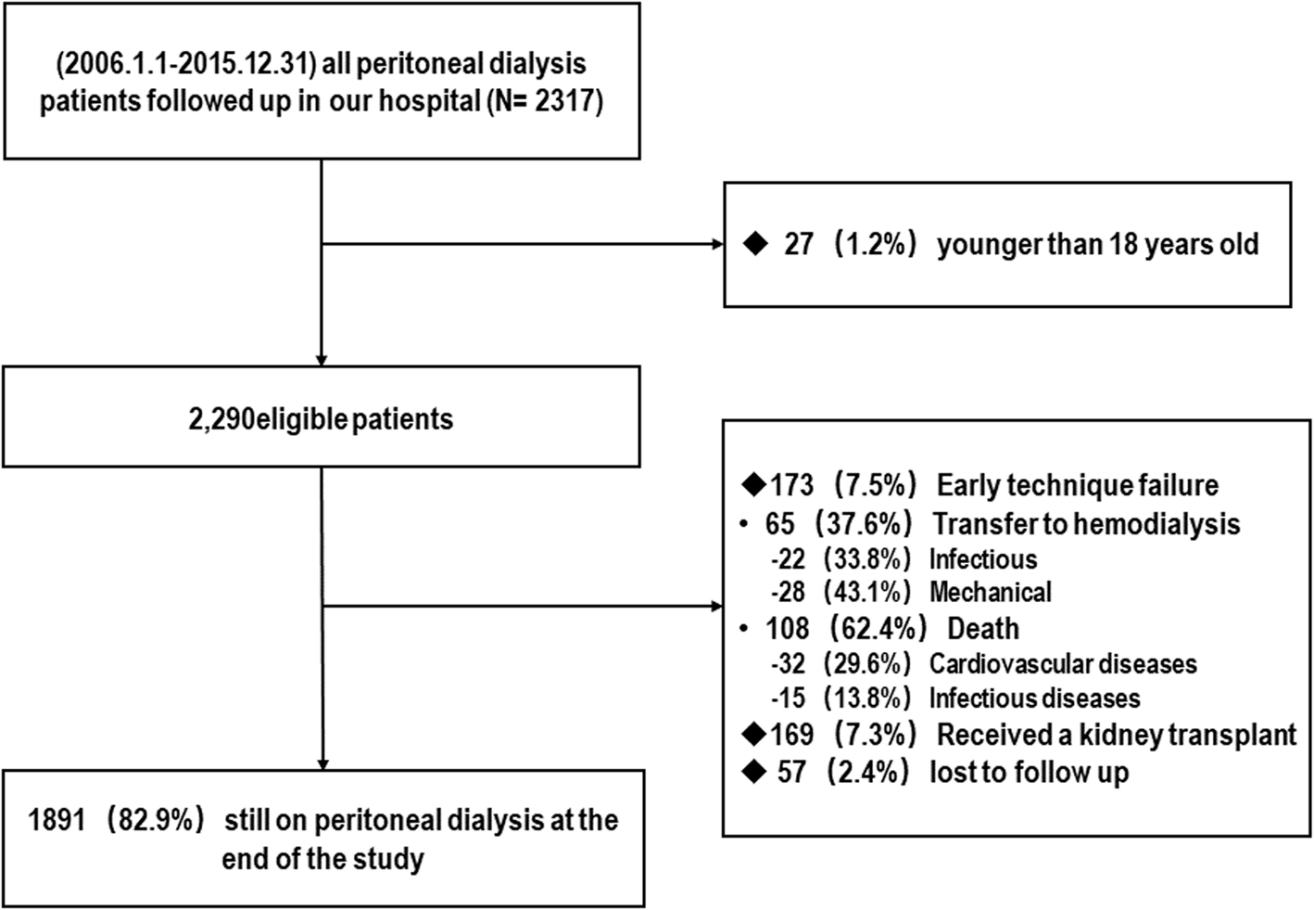
Flow chart of participant selection for this cohort study

A total of 2,317 incident PD patients were follow-up at our PD center, of them 27 patients younger than 18 years were excluded. The remaining 2,290 patients were enrolled in this study (Fig. 1). The mean age was 48.2 ± 15.7 years, 59.1%were male and 25.2% with diabetes. Baseline characteristics of patients with or without technique failure are described in Table 1. During the first year of PD, 108 (4.7%) patients had died, 65(2.8%) patients had transferred to HD, 169 (7.3%) cases received kidney transplantation, and 57 (2.4%) cases had lost to follow-up. The technique failure rate within the first year of PD (including death and transferring to HD) was 7.5% (*n* = 173 patients).

**Table 1:** Baseline Characteristics of Patients Initiated PD in 2006–2015

	**Overall**	**No Technique Failure**	**Technique Failure**	***P value***
	*N*** = 2,290**	**(***n*** = 2117)**	**(***n*** = 173)**	
**Male gender**	1,354(59.1%)	1,252(59.1%)	102(59.0%)	0.84
**Age**				< 0.001
** < 65Y**	1,918(83.8%)	1813(85.6%)	105(60.7%)	
** ≥ 65Y**	372(16.2%)	304(14.4%)	68(39.3%)	
**BMI**				< 0.001
** < 18.5 kg/m**^**2**^	443(19.2%)	396 (18. 6%)	47 (27.2%)	
** 18.5–24 kg/m**^**2**^	1365(59.0%)	1288(60.2%)	77(44.5%)	
** > 24 kg/m**^**2**^	482(21.1%)	433(20. 5%)	49 (28.3%)	
**Education**				0.008
** under the primary **	855(37.3%)	784(3.7%)	71(41.0%)	
** primary**	913(39.9%)	834(39.4%)	79(45.7%)	
** higher**	522(22.8%)	499(23.6%)	23(13.3%)	
**Medical insurance**				< 0.001
** RMI**	1,355(59.2)	1,222(57.7)	133(76.9)	
** EMI**	825(36.0)	790(37.3)	35(20.2)	
** CI or FM**	110(4.8)	105(5.0)	5(2.9)	
**Annual income**				0.22
** ≤ 50 thousand RMB**	1,817(79.3)	1,668(78.8)	149(86.1)	
** > 50 thousand RMB**	473(20.7)	449(21.2)	24(13.9)	
**Self-care ability**				< 0.001
** Not completely**	189(8.25%)	153(7.23%)	36(20.8%)	
** Complete self-care**	2,101(91.6%)	1,964(92.8%)	137(79.2%)	
**Cause of ESKD**				< 0.001
** Glomerulonephritis**	1361(59.4%)	1283(60.6%)	78(45.1%)	
** Diabetes**	492(21.5%)	435(20.6%)	57(33.0%)	
** Hypertension**	171(7.5%)	159(7.5%)	12(6.9%)	
** Other**	261(11.6%)	240(11.3%)	21(15.0%)	
**Comorbidity conditions**				
** Diabetes**	578 (25.2%)	512 (24.2%)	66(38.2%)	< 0.001
** CVD**	539(23.5%)	474(22.4%)	65(37.6%)	0.025
** IHD**	492(21.5%)	441(20.8%)	51(29.5%)	< 0.001
** CHF**	70(3.1%)	45(2.13%)	25(14.5%)	< 0.001
**Laboratory variables**				
** hemoglobin (g/L)**	79.8 ± 19.0	79.7 ± 19.00	82.5 ± 18.6	0.03
** albumin(g/L)**	34.9 ± 4.89	35.0 ± 4.86	33.8 ± 5.06	< 0.001
** Scr (mg/dL)**	10.9 ± 4.35	11.0 ± 4.38	9.71 ± 3.81	< 0.001
** eGFR (mL/min/1.73 m**^**2**^**)**	5.69 ± 2.59	5.63 ± 2.53	6.33 ± 3.09	< 0.001
** calcium (mmol/L)**	1.98 ± 0.28	1.98 ± 0.28	1.97 ± 0.27	0.29
** phosphorus(mmol/L)**	2.04 ± 0.54	1.97 ± 0.27	1.99 ± 0.49	0.38
** I-PTH (pg/ml)**	412 ± 307.3	415 ± 308.4	378 ± 315.1	0.07
** Triglycerides(mmol/L)**	1.55 ± 0.98	1.54 ± 0.97	1.74 ± 1.11	< 0.001
** Total cholesterol(mmol/L)**	4.69 ± 1.39	4.67 ± 1.36	4.86 ± 1.67	< 0.001
** HDL-C (mmol/L)**	1.03 ± 0.32	1.04 ± 0.32	1.01 ± 0.38	0.21
** LDL-C (mmol/L)**	2.84 ± 1.03	2.84 ± 1.03	2.88 ± 1.13	0.78
**Duration of RRT**				< 0.001
** Nil**	854(37.2%)	772(36.6%)	80(46.2%)	
** ≤ 90d**	1355(59.1%)	1273(60.1%)	82(47.4%)	
** > 90d**	82(3.62%)	72(3.40%)	11(6.63%)	
**Peritonitis**	282(12.3%)	240(11.3%)	42(24.8%)	< 0.001

Values are given as number (percentage)and *P* value for each variable

Abbreviations: *PD* Peritoneal dialysis, *RMI* Resident medical insurance, *EMI* Employee medical insurance, *CI or FM* Commercial insurance or Free medical, *I-PTH* intact parathyroid hormone, *Scr* Serum creatinine, *HDL-C* High-density lipoprotein cholesterol, *LDL-C* Low-density lipoprotein cholesterol, *GFR* Glomerular filtration rate, *BMI* Body mass index, *CVD* Cardiovascular disease, *ESKD* End stage kidney disease, *HD* Hemodialysis, *CHF* Congestive heart failure, *IHD* Ischemic heart disease, *PD* Peritoneal dialysis, *RRT* Renal replacement therapy

### The causes of technique failure in the first year of PD

**Fig. 2:**
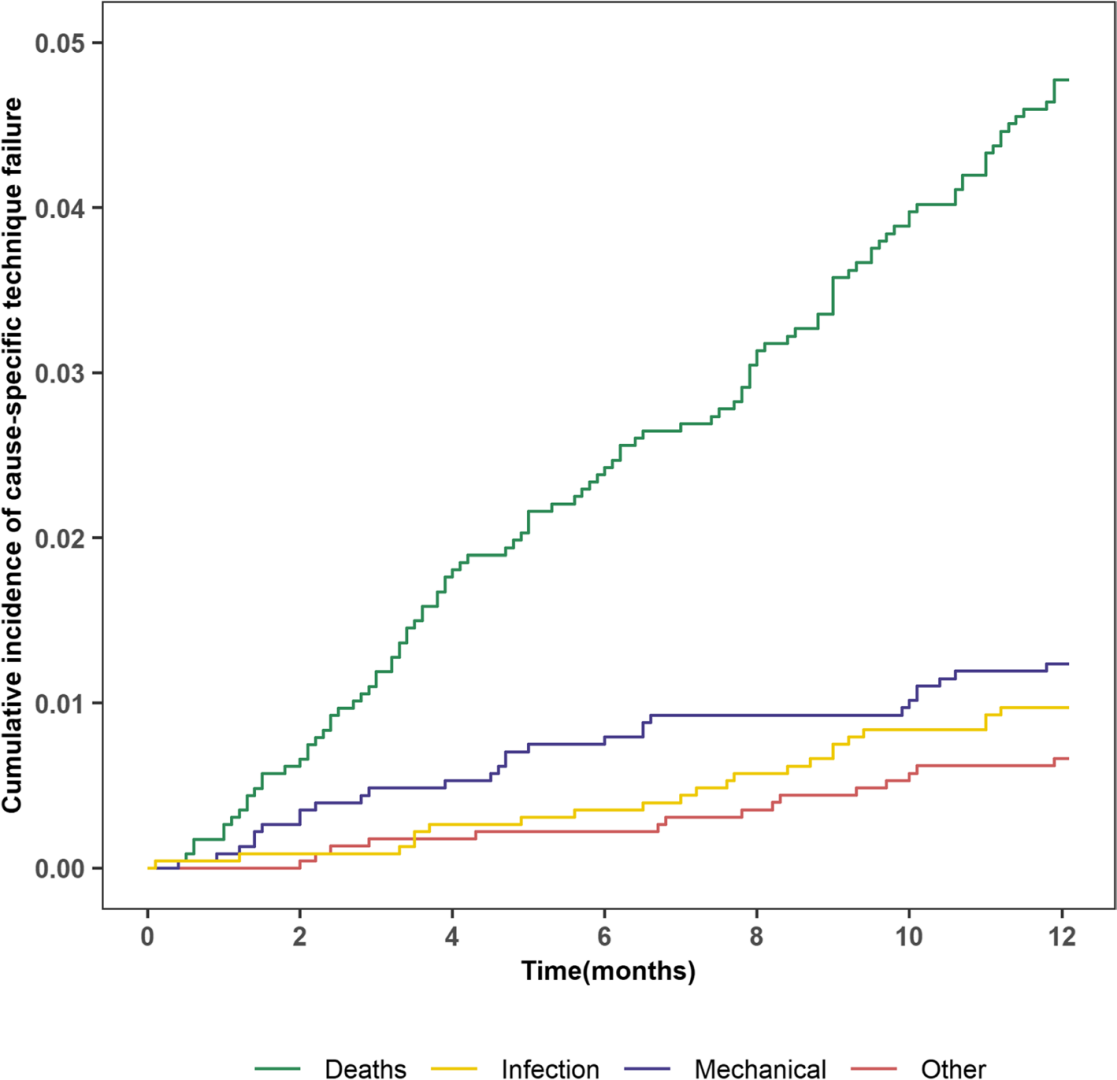
Cumulative incidence of cause-specific technique failure. Curves represent the cumulative incidence of each cause of technique failure, with other causes (death, infectious, mechanical, or other) and transplantation examined as competing risks

Among the 173 cases of technique failure occurred in the first year of PD, death account for 62.4%(*n* = 108) and transferring to HD account for 37.6% (*n* = 65). The main reasons for death were cardiovascular diseases (*n* = 32, 29.6%), infectious diseases (15, 13.8%) and for transferring to HD were mechanical cause (*n* = 28, 43.1%), infection cause (*n* = 22, 33.8%), and other causes (*n* = 15,23.1%) (Fig. 1, Table 1). The risk of technique failure caused by death was constant and obvious during the first year of PD. In the first six months, the incidence of technical failures caused by mechanical problems was much higher than by infection and other reasons (Fig. 2), transfering to HD caused by infection and by mechanical account 9% and 21% of technique failure (Table 2).

**Table 2:** Rates of Technique Failure and Transplantation at the first 6 and 12 Months of PD

	**6 month**	**12 month**
Technique Failure	86(3.7%)	173(7.5%)
Transferring to HD	31(36%)	65(37%)
Transferring to HD caused by Infection	8(9%)	22(12%)
Transferring to HD caused by Mechanical	18(21%)	28(16%)
Transferring to HD caused by Other	5(6%)	15(9%)
Death	55(63%)	108(62%)
Transplantation	90(3%)	169(7%)

Abbreviations: *HD* Hemodialysis

### The risk factors associated with technique failure during the first year of PD

Covariates with *P* < 0.05 in the univariate analyses or thought to be related to the outcome of interest were chosen for multivariate proportional hazards regression. The results shown that advanced age (HR 2.78, 95%CI 1.82–4.30), low body mass index (BMI < 18.5 kg/m^2^: HR 1.77, 95%CI 1.17–2.67), congestive heart failure (HR 2.81, 95%CI 1.58–4.98), time on HD before PD ≤ 3 months (HR 1.49, 95%CI 1.05–2.10), peritonitis (HR 2.02, 95%CI 1.36–3.01) were associated with the increased risk of technique failure.; while higher serum albumin (HR 0.93, 95%CI 0.89–0.96) and using employee medical insurance to pay medical expenses (HR 0.47, 95%CI 0.32–0.69) were associated with reduced risk. (Table 3).

**Table 3:** Multivariable Competing-Risk Regression Analysis of Technique Failure And Cause-Specific Technique Failure Within First-Year of PD

	**Technique Failure**	**P**	**Death**	**P**	**Transfer to HD**	**P**
	**SHR (95% CI)**		**SHR (95% CI)**		**SHR (95% CI)**	
**Male gender**	1.06(0.70–1.63)	0.76	1.13(0.66–1.93)	0.65	1.05(0.50–2.20)	0.90
**Age**						
** < 65Y**	Reference		Reference		reference	
** ≥ 65Y**	2.78(1.82–4.30)	< 0.001	4.36(2.46–7.74)	< 0.001	1.22(0.53–2.34)	0.76
**BMI**						
** < 18.5 kg/m**^**2**^	1.77(1.17–2.67)	0.01	2.42(1.44–4.06)	< 0.001	0.98(0.46–2.10)	0.96
** 18.5–24 kg/m**^**2**^	Reference		Reference		reference	
** > 24 kg/m**^**2**^	1.46(0.98–2.14)	0.06	1.38(0.82–2.35)	0.22	1.45(0.81–2.60)	2.06
**Education**						
** under the primary**	Reference		Reference		reference	
** primary**	1.18(0.81–1.70)	0.39	1.03(0.64–1.64)	0.91	1.43(0.76–2.66)	0.26
** higher**	0.77(0.46–1.28)	0.31	0.73(0.38–1.40)	0.35	0.78(0.32–1.91)	0.60
**Medical insurance**						
** RMI**	Reference		Reference		Reference	
** EMI**	0.47(0.32–0.69)	< 0.001	0.41(0.24–0.70)	< 0.001	0.55(0.31–0.99)	0.05
** CI or FM**	0.38(0.15–1.01)	0.05	0.41(0.14–1.24)	0.12	0.34(0.05–2.58)	0.30
**Self-care ability**						
** not completely**	Reference		Reference		reference	
** complete**	0.67(0.43–1.05)	0.08	0.53(0.32–0.88)	0.02	1.41(0.50–3.99)	0.51
**Cause of ESKD**						
** Glomerulonephritis**	Reference		Reference		reference	
** Diabetes**	0.66(0.35–1.27)	0.21	0.58(0.26–1.30)	0.17	0.96(0.32–2.89)	0.95
** Hypertension**	0.80(0.38–1.67)	0.54	0.70(0.26–1.92)	0.49	1.01(0.36–2.86)	0.97
** Other**	1.43(0.88–2.41)	0.17	1.30(0.64–2.65)	0.47	1.56(0.75–3.24)	0.23
**Comorbidity conditions**						
** Diabetes**	1.52(0.86–2.68)	0.15	1.93(0.95–3.92)	0.07	0.97(0.34–2.75)	0.99
** CVD**	1.57(0.84–2.94)	0.16	1.72(0.85–3.49)	0.13	1.37(0.38–4.95)	0.63
** IHD** ** CHF**	0.67(0.36–1.24) 2.81(1.58–4.98)	0.20 < 0.001	0.57(0.29–1.08) 3.52(1.83–6.75)	0.09 < 0.001	1.10(0.28–4.26) 0.81(0.12–5.40)	0.88 0.83
**Laboratory variables**						
** Hemoglobin(g/L)**	1.01(0.99–1.02)	0.17	1.01(0.99–1.02)	0.15	1.00(0.98–1.02)	0.93
** Albumin(g/L)**	0.93(0.89–0.96)	< 0.001	0.92(0.88–0.96)	< 0.001	0.95(0.90–1.00)	0.67
** Total cholesterol (mmol/L)**	0.96(0.83–1.12)	0.63	1.01(0.85–1.19)	0.93	0.92(0.70–1.21)	0.53
** Triglycerides (mmol/L)**	0.95(0.85–1.07)	0.42	1.10(0.88–1.36)	0.39	0.86(0.70–1.07)	0.18
** Scr(mg/dL)**	0.99(0.91–1.08)	0.89	0.99(0.89–1.11)	0.88	1.00(0.89–1.14)	0.92
** eGFR(mL/min/1.73m**^**2**^)	1.02(0.92–1.13)	0.76	0.98(0.85–1.12)	0.77	1.09(0.93–1.27)	0.33
**Duration of RRT**						
** 0**	Reference		Reference		reference	
** > 90**	1.20(0.50–2.89)	0.68	1.36(0.45–4.05)	0.59	1.07(0.26–4.47)	0.92
** ≤ 90**	1.49(1.05–2.10)	0.03	1.68(1.05–2.69)	0.03	1.29(0.75–2.23)	0.36
** Peritonitis**	2.02(1.36–3.01)	< 0.001	1.10 (0.61–1.94)	0.77	3.95(2.25–6.80)	< 0.001

*N *= 2,290

Transplantation, death and transfer to HD was competing risk respectively

Abbreviations: *PD* Peritoneal dialysis, *RMI* Resident medical insurance, *EMI* Employee medical insurance, *CI or FM* Commercial insurance or Free medical, *I-PTH* intact parathyroid hormone, *Scr* Serum creatinine, *HDL-C* High-density lipoprotein cholesterol, *LDL-C* Low-density lipoprotein cholesterol. *GFR* Glomerular filtration rate, *BMI* Body mass index, *CVD* Cardiovascular disease, *ESKD* End stage kidney disease, *HD* Hemodialysis, *CHF* Congestive heart failure, *IHD* Ischemic heart disease, *PD* Peritoneal dialysis, *RRT* Renal replacement therapy

### The risk factors associated with technique failure caused by death or by transferring to HD

The results of multivariate analysis showed that advanced age (≥ 65 years old: HR 4.36, 95%CI 2.46–7.74), lower BMI (BMI < 18.5 kg/m^2^: HR 2.42, 95%CI 1.44–4.06) and time on HD before PD ≤ 3 months (HR 1.68, 95%CI 1.05–2.69), congestive heart failure (HR 3.52, 95%CI 1.83–6.75) were associated with an increased risk of death. Complete self-care ability (HR 0.53, 95% CI 0.32–0.88), using employee medical insurance to pay medical expenses (HR 0.41, 95%CI 0.24–0.70), and higher serum albumin (HR 0.92, 95%CI 0.88–0.96) were associated with a decreased risk of death in the first year of PD (Table 3).

For the technique failure due to transferring to HD, PD-related peritonitis was associated with the increased risk (HR 3.95, 95%CI 2.25–6.80), while using employee medical insurance to pay medical expenses was associated with reduced risk (HR 0.55, 95%CI 0.31–0.99) in the first year of PD (Table 3).

## Disussion

In this retrospective cohort study, we analyzed the incidence and risk factors of the technique failure in the first year of PD on 2,290 incident PD patients. Overall, the incidence of technique failure within the first year after the start of PD was 7.5%. The rate of technique failure due to death was 4.7% and due to transferring to HD was 2.8%. The main causes of early death were cardiovascular diseases and infectious diseases, while the main causes of early transfering to HD were mechanical failure and infection. Advanced age, lower BMI, history of HD or congestive heart failure and peritonitis were the factors associated with increased risk for the technique failure, while using employee medical insurance to pay expenses and high serum albumin associated with decreased risk for the technique failure.

In a study involving more than 30,000 patients in the United States, the rate of transferring to HD during 1 year reached 18.7%-20.5% [10]. ANZDATA Study on 16,748 PD patients reported that 4,389 patients (26.2%) developed early technique failure (including death and transferring to HD) during the first year of PD therapy. In a study of Singapore, 19% of patients developed to technique failure (including death and transferring to HD) in first year after PD start [11]. A retrospective cohort study of 5,162 PD patients in Canadian showed that the 1-year conversion rate to HD was 12.7% [12]. In current study, the technique failure rate within the first year of PD in our center was 7.5%. The relative low incidence of technique failure in the early stage of PD may attribute to the Asian race, younger cohort, less comorbidity and larger PD center size [13, 14]. More than 1,000 PD patients have been followed up by a well-trained PD team in our center since 2012. A unique therapy and management approach that includes a standardized procedure for catheter insertion, a carefully designed PD prescription, a meticulous and comprehensive patient training and follow-up care may involve in the lower technique failure rate [15].

In our cohort, 25.2% of patients had diabetes and 23.5% had cardiovascular disease. In a study of 725 ESRD patients enrolled in 36 dialysis centers in China, the average age of the cohort was 49.8 years old, only 16.3% had a history of cardiovascular disease and 27.8% had diabetes, and the main cause of ESRD was chronic glomerulonephritis [16]. These results confirmed the baseline characteristics of lower age and low co-morbidity in China, which are much different from the data of other western countries. In the 2021 annual report of the US Kidney data system, the average age of ESRD patients was 62.7 years, 66.2% had cardiovascular disease and 60.6% had diabetes [17]. Chronic glomerulonephritis, which always occur in young population, is the main cause of ESRD in China. The high incidence of hypertension among this population may increases the incidence of CVD in the cohort [18]. In a national cross-sectional study of 47,204 CKD patients in China, the incidence of hypertension was 60.5%, significantly higher than that of diabetes (19.1%) and CVD (9.5%) in the participants with eGFR < 60 mL/min per 1·73 m^2^ [19]. These may explain the reasons of high incidence of CVD and low diabetes in this cohort.

We demonstrated that death was the leading cause of the technique failure. Among the 173 cases of technique failure occurred in the first year of PD, death account for 62.4%. In a Dutch study, deaths accounted for 69% of the total number of early technique failures [20]. Studies in South Korea, Singapore and Canada also demonstrated that death was the leading cause of early withdrawal from PD [11, 12, 21]. In our study, cardiovascular death was the leading cause of death (29.6%). We found that a baseline history of congestive heart failure was also a risk factor for death and technique failure. According to recent reports, patients with volume overload at the baseline have a higher risk of death and conversion to HD over the next 12 months [22]. The early history of heart failure often indicates poor volume control, poor residual renal function and cardiac function, insufficient ultrafiltration and peritoneal permeability, and poor effect of PD model or treatment prescription [23–25]. The control and monitoring of patient volume and maintenance of patient's fluid balance are very important for patient's technical survival. According to our previous report, about 86.5% of the catheter function problems occurred in the first year of PD [26]. In the current study, the results showed that transferring to HD due to mechanical problem accounted 16.2%, of which 64.0% occurred in the first six months. RDPLF study revealed that catheter dysfunction accounted for 18.1% of the early conversion to HD [5]. In ANZDATA study, mechanical causes accounted for 19.7% of the early technique failure (including death and transferring to HD) [3]. All these studies have similar results which indicated mechanical problem played an important role in the technique failure due to the transferring to HD during the early stage of PD.

Our previous study demonstrated that early peritonitis was a risk factor for early death-censored technique failure [27]. The current study also demonstrated that peritonitis was not only a cause but also a risk factor associated with technique failure during the first year of PD [28]. ANZDATA reported that the incidence of direct conversion to HD in new PD patients due to peritonitis within the first 12 months was high as 16.4% [29]. RDPLF study also showed that patients with early peritonitis had a 53% increased risk of transferring to HD [30]. A cohort from South Africa revealed that the risk of technique failure in patients with more than one attack of peritonitis increased by 90% [31]. In addition, studies have shown that peritonitis was also associated with an increased risk of all-cause death in PD patients [32, 33]. A recent study in our center found that the impact of peritonitis on mortality was more significant in patients with longer PD duration[34]. However, there was no direct evidence that peritonitis increased the risk of death in the early stage of PD.

The current study revealed that elderly patients was significantly associated with technique failure, in particularly, the death, but not with transferring to HD in the first year of PD. ANZDATA's study demonstrated that age > 70 was an independent risk factor for early technique failure (including death) in PD, with a 43% higher risk compared to younger PD patients [3]. It has been documented that history of HD was a risk factor for technique failure in previous studies [3, 5, 12]. Here we also demonstrated that patients with a previous history of HD had an increased risk of early technique failure. In addition, our study found that PD patients with complete self-care have a lower risk of death during the first year of PD. McGill, et al. reported that the risk of death of working patients decreased by 18% and the risk of conversion to HD decreased by 27% [35]. Patients with old age, previous history of HD or frailty have the problems that may affect their compliance to PD treatment and therefore affect their prognosis [36]. Attention should be paid for the special population to reduce the technique failure in the early stage of PD.

Both a lower albumin levels and BMI reflect malnutrition. It was well documented that hypoproteinemia was associated with an increased risk of deaths as well as peritonitis [37]. Our previous study demonstrated that patients with serum albumin < 3.5 g/dL had a 75% increased risk of peritonitis [27]. A study from the Middle East found that a dynamic decrease in serum albumin in PD patients indicated an increased risk of transferring to HD [38]. The current results further demonstrated that lower serum albumin was associated with early death and technique failure. ANZDATA's study revealed that lower BMI (< 18.5 kg/m^2^) increases the risk of early technique failure compared with BMI (18.5-30 kg/m^2^) in the first-year of PD [3]. However, higher BMI in the study of Jaar et al. was reported to be associated with the risk of early transferring to HD [39]. Our study showed that low BMI were associated with early technique failure, and that low BMI was significantly associated with death.

We did not find the relationship between diabetes and early technique failure. Previous studies have shown that diabetes significantly increased the risk of technique failure [13, 20]. ANZDATA have found that diabetes was risk factor for technique failure within one year [3]. Previous studies on patients who withdrew from PD (including death, HD and renal transplantation) within an ultra-early stage (3 months) did not find that diabetes was associated with the drop-out from PD [40]. The effect of education levels on early technique failure was unclear. A study from South Korea reported that patients in junior high school and below were associated with early technique failure [41]. Chidambaram's study found that the level of education in the place of residence was related to the risk of early technique failure [12]. However, we did not demonstrate the effect of education level on early technique failure.

In a national study in South Korea, patients who participated in comprehensive health insurance had a relatively low risk of technique failure [21]. Our study revealed that patients with employee medical insurance had a lower risk of death and transferring to HD than patients with resident medical insurance. It is reported that the China medical insurance coverage rate of registered residents in 2011 is 95.7% [42]. Among them, residents' medical insurance is the main body of Guangzhou medical system, which is jointly borne by residents and government finance, and the insured objects include minors, unemployed residents and elderly residents. However, due to China's strict household registration system, non-Guangzhou household registration patients need to return to their domicile for reimbursement. Urban workers' medical insurance is mainly open to employees of government units and state-owned enterprises and employees of some private enterprises, and is jointly borne by employers, employees and government finance (about 6% of the total wages paid by employers and 2% of employees' monthly wages) [43]. The reimbursement scope of employee medical insurance is wider than that of resident insurance [44]. In addition, patients who can participate in employee health insurance tend to have better socio-economic adjustment.

Our current investigation has several limitations. First of all, this is a retrospective cohort study that the evidence of causality is not strong. Secondly, due to the large time span of this study, many covariates were not included in the analysis, such as peritoneal function, catheter selection and operation, psychological status, subjective indicators of patients and doctors, and so on. In addition, due to the limitations of the level of economic development, China's APD and icodextrin are still not widely available and therefore are not included in the analysis. Third, factors related to the characteristics of the center are not included in this single-center study, such as PD prescription habits, anti-infective regimen, center size, etc. However, it is worth noting that we identified the risk factors associated with technique failure during the first year of PD based on demographic and laboratory data, which were not available in previous studies.

In summary, this retrospective cohort study on 2,290 incident PD patients in Southern China revealed incidence of technique failure within the first year of PD was 7.5%. Advanced age, low BMI, history of congestive heart failure or time on HD before PD ≤ 3 months and peritonitis were the factors associated with increased risk, while use employee medical insurance and higher serum albumin associated with decreased risk for the technique failure. Identification of the causes and risk factors may be helpful for the PD center to improve its medical plan and reduce incidence of the technique failure in the early stage of PD. Further studies are needed to clarify the underlying mechanism.

## Supplementary Information

**Additional file 1.**

## Data Availability

We have put the raw data in the supplementary file. All data generated or analyzed during this study are included in this published article and its supplementary information files.
